# New allele of *C. elegans* gene *spch-3 (T27A3.4)*, called *xc2*

**DOI:** 10.17912/W2995W

**Published:** 2018-05-01

**Authors:** Nicholas R. Munoz, Dana T. Byrd, Diana Chu

**Affiliations:** 1 Department of Biology, San Francisco State University, San Francisco, California 94132, USA

**Figure 1.**
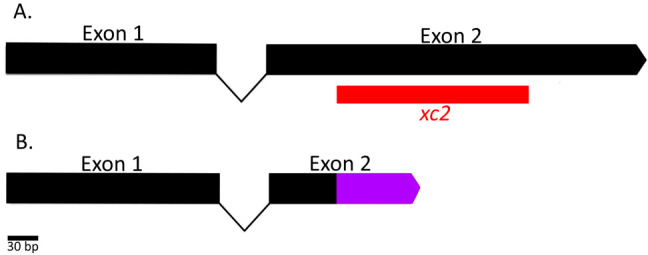
**Figure 1.** A) Map of the exons, and intron of *spch-3* (T27A3.4) with the location of the *xc2* deletion in red. B) Map of the new *spch-3* allele after mutation, with frameshifted nucleotides to the first stop codon in purple.

## Description

We created a new mutant allele, named *xc2,* of the gene *spch-3* (T27A3.4). *spch-3* encodes one of three *C. elegans* small, highly basic proteins that resemble invertebrate protamines and associate with sperm meiotic chromosomes (Chu et al., 2006). The alleles were isolated from gene mutations generated by Non-Homologous End Joining (NHEJ) mediated repair of Cas9-generated breaks (Dickinson et al., 2013; Ran et al., 2013). The alleles were discovered using PCR with the following primers 5’- CCCTCGTCTTCTCTACTAGA -3’ and 5’- CATAGCTTCACAGGGAGAAG -3’. Next Generation Sequencing let us identify 30 bp flanking sequences of the *xc2* allele as GTCCGTCAACCCGCCGTTCGTCGTCTCGTC – [191 bp deletion] – TCTCGTCATCGCTCATCTTCGAAGGCTCGT. Wildtype *spch-3* has 2 exons. The *xc2* mutation is located in the second exon (Fig. 1A). In the mutant, while the first 97 amino acids of the mutant protein remain wildtype, they are followed by the deletion causing a frameshift mutation that adds 25 mutant amino acids (LSSSLIFEGSWTSCVDCKEDSCQKT) before the first resulting stop codon (Fig. 1B). Therefore, out of the 203 amino acids in the wildtype SPCH-3 protein, only the first 97 remain in the *xc2* allele. Although the additional 25 amino acids may have an effect that is yet to be determined, the mutation in the second exon changes about 52% of the protein providing a unique opportunity to understand the function of SPCH-3 through mutant studies.

## Reagents

Strains: **XC111** *spch-3(xc2)*I (outcrossed 5x)
